# Bazi Bushen ameliorates age-related energy metabolism dysregulation by targeting the IL-17/TNF inflammatory pathway associated with SASP

**DOI:** 10.1186/s13020-024-00927-9

**Published:** 2024-04-09

**Authors:** Xiaogang Shen, Mengnan Li, Yawen Li, Yuning Jiang, Kunxu Niu, Shixiong Zhang, Xuan Lu, Runtao Zhang, Zhiqin Zhao, Liangxing Zhou, Zhifang Guo, Siwei Wang, Cong Wei, Liping Chang, Yunlong Hou, Yiling Wu

**Affiliations:** 1 Hebei Medical University, Hebei Province, 361 East Zhongshan Road, Shijiazhuang, 050017 People’s Republic of China; 2 National Key Laboratory for Innovation and Transformation of Luobing Theory, Shijiazhuang, 050035 People’s Republic of China; 3 Nanjing University of Chinese Medicine, Nanjing, 210023 People’s Republic of China; 4 Hebei University of Chinese Medicine, Shijiazhuang, 050091 People’s Republic of China; 5 High-level TCM Key Disciplines of National Administration of Traditional Chinese Medicine—Luobing Theory, Hebei Yiling Hospital, Shijiazhuang, 050091 Hebei Province People’s Republic of China; 6 Shijiazhuang New Drug Technology Innovation Center of Compound Traditional Chinese Medicine, Shijiazhuang, 050035 People’s Republic of China

**Keywords:** Bazi Bushen (BZBS), Energy metabolism, Omics analysis, Lipid metabolism, Inflammation

## Abstract

**Background:**

Chronic inflammation and metabolic dysfunction are key features of systemic aging, closely associated with the development and progression of age-related metabolic diseases. Bazi Bushen (BZBS), a traditional Chinese medicine used to alleviate frailty, delays biological aging by modulating DNA methylation levels. However, the precise mechanism of its anti-aging effect remains unclear. In this study, we developed the Energy Expenditure Aging Index (EEAI) to estimate biological age. By integrating the EEAI with transcriptome analysis, we aimed to explore the impact of BZBS on age-related metabolic dysregulation and inflammation in naturally aging mice.

**Methods:**

We conducted indirect calorimetry analysis on five groups of mice with different ages and utilized the data to construct EEAI. 12 -month-old C57BL/6 J mice were treated with BZBS or β-Nicotinamide Mononucleotide (NMN) for 8 months. Micro-CT, Oil Red O staining, indirect calorimetry, RNA sequencing, bioinformatics analysis, and qRT-PCR were performed to investigate the regulatory effects of BZBS on energy metabolism, glycolipid metabolism, and inflammaging.

**Results:**

The results revealed that BZBS treatment effectively reversed the age-related decline in energy expenditure and enhanced overall metabolism, as indicated by the aging index of energy expenditure derived from energy metabolism parameters across various ages. Subsequent investigations showed that BZBS reduced age-induced visceral fat accumulation and hepatic lipid droplet aggregation. Transcriptomic analysis of perirenal fat and liver indicated that BZBS effectively enhanced lipid metabolism pathways, such as the PPAR signaling pathway, fatty acid oxidation, and cholesterol metabolism, and improved glycolysis and mitochondrial respiration. Additionally, there was a significant improvement in inhibiting the inflammation-related arachidonic acid-linoleic acid metabolism pathway and restraining the IL-17 and TNF inflammatory pathways activated via senescence associated secretory phenotype (SASP).

**Conclusions:**

BZBS has the potential to alleviate inflammation in metabolic organs of naturally aged mice and maintain metabolic homeostasis. This study presents novel clinical therapeutic approaches for the prevention and treatment of age-related metabolic diseases.

**Supplementary Information:**

The online version contains supplementary material available at 10.1186/s13020-024-00927-9.

## Introduction

Aging is a natural process that involves various physiological changes, with one of the most significant impacts being the marked decline in metabolic pathways, especially energy metabolism, in later life stages [1]. Aging affects glucose and lipid metabolism by modifying the distribution and function of adipose tissue, reducing the metabolic activity of brown and beige adipocytes, impairing insulin sensitivity and secretion, and altering the hormonal and nutritional signals that regulate energy metabolism [2–6]. Dysfunctions in glucose and lipid metabolism can result in different chronic metabolic diseases, including obesity, type 2 diabetes, cardiovascular disease, and fatty liver disease [7–9]. These diseases are linked to elevated inflammation, oxidative stress, cellular senescence, and epigenetic modifications, all of which can accelerate aging and raise the risk of aging-related diseases, including cancer, neurodegeneration, and frailty [10–13]. Although the association between aging and metabolic decline is well-established, the precise mechanisms and causal links remain a complex and continuously evolving area of research.

With aging, our bodies accumulate senescent cells, which are cells that have ceased dividing but remain metabolically active [14]. These cells release pro-inflammatory factors such as cytokines and chemokines, resulting in a persistent inflammatory environment that disrupts normal metabolism [13, 15] Inflammatory mediators convert unaffected by standers, such as liver cells, into fat storage depots, while concurrently impairing insulin signaling in muscle and adipose tissue [16–18]. On the other hand, the ability of organs to utilize lipids as energy substrates is reduced, thus further aggravating “ectopic lipid accumulation” [19]. Lipid deposition in tissues can lead to glucose intolerance and other metabolic complications, exacerbating inflammation [20, 21]. Consequently, chronic inflammation and metabolic dysfunction create a vicious cycle, accelerating aging and increases the risk of chronic diseases. Mounting evidence indicates that this chronic, low-grade inflammation,—also known as “inflammaging”,—is a key factor that disrupts the intricate balance between glucose and lipid metabolism [22, 23].

While the development of inflammaging-targeted therapies is still in its early stages, the potential is immense. Several promising approaches are being explored to target inflammaging and promote healthy aging. For instance, senolytics, a type of medicines that selectively eliminating senescent cells, can reduce the source of chronic inflammation [24]. Preclinical studies in mice have demonstrated that senolytics improved insulin sensitivity and reduced fat accumulation in the liver, potentially leading to beneficial effects on metabolic health [25]. Furthermore, long-term application of senolytics in mice can improve physical function, reduce inflammation, and potentially extend lifespan [26]. Natural flavonoids are notably an important class of representative compounds among all senolytics. Early clinical trials suggest that flavonoids have the potential to reduce senescent cell burden by inducing apoptosis in these cells and improving physical function in older adults [27]. Furthermore, various natural compounds and medications with anti-inflammatory properties are currently under investigation for their potential to alleviate inflammaging. These include omega-3 fatty acids, curcumin, and resveratrol [28–32]. These findings clearly demonstrate the following: (1) targeting inflammaging shows great potential for anti-aging purposes; (2) targeting inflammaging provides a glimmer of hope for transforming our approach to age-related metabolic dysfunction and chronic diseases; (3) Active ingredients derived from natural plants hold high development value in combating aging and age-related diseases. Despite the significant promise of targeting inflammaging for promoting anti-aging effects, there are still several challenges that need to be addressed. Ongoing research and development efforts are dedicated to enhancing targeting specificity, ensuring long-term safety, improving delivery methods, and minimizing off-target effects to achieve safer and more effective senolytic therapies.

**Table 1:** Primer sequences for genes used in qRT-PCR

	Forward primer	Reverse primer
*Actb*	CTATTGGCAACGAGCGGTTC	ACTGTGTTGGCATAGAGGTCTT
*Cxcl1*	GCTGGGATTCACCTCAAGAACATC	GTGTGGCTATGACTTCGGTTTGG
*Cyp2b10*	CCAACCAGCACACGGAGTTC	GCATGAGCAGGAAGCCATAGC
*Fos*	TGACTGGAGGTCTGCCTGAGG	CACGTTGCTGATGCTCTTGACTG

As the anti-aging research field advances, Traditional Chinese Medicine (TCM) has gained significant attention and recognition for its extensive history of research, development, and clinical application in anti-aging practices. BZBS is a Chinese-patented drug. Mass spectrometry analysis has revealed that the main components of BZBS are neochlorogenic acid, chlorogenic acid, cryptochlorogenic acid, isoquercitrin, hyperoside, verbascoside, epimedin A, icariin, baohuoside I, imperatorin, osthole, catalpol, deoxyschizandrin, and schisandrin B [33]. Experimental evidence has shown that neochlorogenic acid can down-regulate genes involved in fatty acid and cholesterol synthesis, up-regulate genes involved in fatty acid β-oxidation, and prevent lipid accumulation in hepatic cells [34]. Chlorogenic acid can up-regulate genes involved in fatty acid oxidation, down-regulate genes involved in triglyceride synthesis and fatty acid transport and reduce hepatic lipid accumulation in diabetic db/db mice; it also decreases the expression of pro-inflammatory cytokine genes in the liver [35]. Cryptochlorogenic acid has been observed to improve lipid peroxidation accumulation and pancreatic islet damage in both in vivo and in vitro diabetes models in a concentration-dependent manner [36]. Hyperoside has been found to significantly improve hepatic steatosis, insulin resistance, and inflammatory response in high-fat diet-induced non-alcoholic fatty liver disease (NAFLD) [37]. Verbascoside plays a role in the development of an atherosclerosis model by regulating glycerophospholipid metabolism in the liver of Western diet-fed apolipoprotein E-deficient mice [38]. These studies provide valuable clues to the pharmacological mechanisms of BZBS in exerting anti-metabolic aging effects. Previous studies have demonstrated that high-dose BZBS (high-fat diet + 2.8 g/kg/day BZBS via intragastric injection) can reduce atherosclerotic plaque area in Ovx/ApoE^−/−^ mice (ApoE^−/−^ mice that underwent ovary ligation and bilateral ovariectomy to induce surgical menopause) by improving lipid metabolism and tissue inflammation [33, 39]. Additionally, Mao et al.'s experiments indicated that high-dose BZBS (2.0 g/kg/day BZBS via intragastric injection) can alleviate the liver aging phenotype by regulating liver DNA methylation in 15-month-old mice [40]. Currently, however, the metabolic regulatory effects of this medicine on organs such as the liver and body fat during the aging process remain uncertain.

This study investigated the energy metabolism in C57BL/6 J mice across five different age groups. Additionally, it developed an Energy Expenditure Aging Index (EEAI) to predict biological age and evaluate the anti-aging efficacy of BZBS, making it the first study to do so. Using techniques such as metabolic cage observation, micro-CT detection, and omics analysis, the study comprehensively investigated and confirmed the regulatory effects of BZBS on energy metabolism, glycolipid metabolism, and inflammaging in naturally aging mice. These findings provide valuable insights that can contribute to the prevention and treatment of age-related metabolic diseases.

## Materials and methods

### Animals

Male C57BL/6 J mice were obtained from Sibeifu (Beijing) Biotechnology Co., Ltd. and housed in the SPF Animal Center of Hebei Yiling Pharmaceutical Research Institute. The mice had ad libitum access to food and water. The housing conditions were as follows: 22 ± 1 °C, 55 ± 5% humidity, and a 12-h light–dark cycle. This study was approved by the Animal Ethics Committee of Hebei Yiling Pharmaceutical Research Institute (Ethics number N2021165, N2021169). All animals were handled according to the Guide for the Care and Use of Laboratory Animals published by the National Institutes of Health (NIH Publication No. 80–23, revised in 1996).

Male C57BL/6 J mice (12-month-old) were randomly divided into the following three groups: Normal diet group; BZBS diet group (35 g of BZBS ultrafine powder mixed into each kg of normal diet, equivalent to an average daily intake of 2 g/kg/day for mice; BZBS sourced from Yiling Pharmaceutical Co., Ltd. Lot No. SYB2112001); NMN diet group (3.5 g of NMN mixed into each kg of normal diet, equivalent to an average daily intake of 0.2 g/kg/day for mice; NMN sourced from Bontac Bio-engineering Co., Ltd., China, Lot No. BT05N120A005). All of the medicated diet was prepared by Synergistic Medical Bioengineering Co., Ltd. After 8 months of administration, indirect calorimetry was performed. Male C57BL/6 J mice at 5 months of age were used as the young control group. The chemical analysis of BZBS was previously performed using ultra-performance liquid chromatography/mass spectrometry (UPLC/MS) [33]. We conducted daily monitoring of the remaining weight of the mouse diet, ensuring consistent food intake across all mouse groups.

### Indirect calorimetry

Indirect calorimetry was performed to assess energy expenditure. Male C57BL/6 J mice at 6, 20, 56, 64 and 80 weeks of age (3–4 mice per group) were used for metabolic trajectory delineation. Metabolic parameters were measured using the Phenomaster system (TSE Systems Inc.). The metabolic cage environment was set at 25 °C, 55% humidity, and a 12-h light–dark cycle. After approximately 48 h of adaptation, data from a 72-h period were collected for subsequent analysis. During the measurement period, the mice had ad libitum access to food and water. The light–dark cycle was set at a 12-h light-12-h dark cycle (lights on at 7:00 AM). The measurement interval was set at every 18 min for the measurement of metabolic parameters such as oxygen consumption (VO_2_), carbon dioxide production (VCO_2_), respiratory exchange ratio (RER), and energy expenditure.

### Construction of energy expenditure aging index (EEAI)

EEAI, as a novel biological age assessment index, is used to evaluate the intervention factors that accelerate or delay the aging phenotype of energy expenditure during the natural aging process. The basic requirement for applying EEAI is that each individual has the same optimal circadian rhythm cycle (approximately 1423 min). The construction steps are as follows: (1) Select the energy metabolism parameters recorded continuously for 72 h and calculate the daily average of energy metabolism parameters for each mouse (including VO_2_, VCO_2_, RER, energy expenditure (EE), XT (the number of times the X-axis infrared light is interrupted by all activities of the mouse) + YT (the number of times the Y-axis infrared light is interrupted by all activities of the mouse), Z (the number of times the Z-axis infrared light is interrupted by all activities of the mouse), DistD (the difference in the movement distance), Speed, Drink, Feed, SumR + L (the sum of the number of left and right turns of the mouse running wheel); (2) Employ R software (https://www.r-project.org/; Version: 4.2.2) and RStudio (https://posit.co/products/open-source/rstudio/; Version: 2023.06.1 + 524) to load the TSA (https://stat.uiowa.edu/~kchan/TSA.htm; Version: 1.3.1) package for calculating the top 5 circadian rhythm cycles for each mouse; load the MetaCycle (https://CRAN.R-project.org/package=MetaCycle; Version: 1.2.0) package to calculate the values of ARS-adjphase, ARS-amplitude, meta2d-Base for each mouse’s EE; (3) Use the vegan package (https://github.com/vegandevs/vegan; Version: 2.6-4) in R software to create principal coordinates analysis (PCoA) plots for mice in different age groups; 4) Utilize the randomForest package (https://www.stat.berkeley.edu/~breiman/RandomForests/; Version: 4.7-1.1) in R software to assess the importance of metabolic parameters: Weight, Adjphase (adjusted phase values of mean daily energy expenditure), VO_2_, EE, RER, VCO_2_, Z, Feed, Amplitude (amplitude values of mean daily energy expenditure), XT + YT, Drink, DistD, SumR + L, and Speed. Select the top 6 important predictors based on the IncNodePurity index for optimization of the random forest regression model (performed with 5 repetitions of tenfold cross-validation). The training and testing set of the regression model are set to 70% (n = 11) and 30% (n = 6) of the total dataset, respectively; (5) Utilize the optimized random forest model to calculate the predicted age of each mouse after administration, which is used in constructing the EEAI.

### Body composition analysis

Body composition analysis was performed according to the previously described method to assess body fat, body lean, subcutaneous adipose tissue (SAT), visceral adipose tissue (VAT), and VAT/SAT ratio [41]. In order to avoid the influence of body weight, we selected mice with a body weight close to 37 g in the aged group and drug administration group. After isoflurane anesthesia, body fat and lean tissue volumes of C57BL/6 J mice were measured using a Micro-CT scanner (Perkin Elmer Quantum GX2). The scanning parameters were set as follows: voltage (kV) 90, current (μA) 88, FOV acquisition 72 Recon 72, and scanning mode was standard whole-body 2mx3. The obtained three-dimensional structures from micro-CT and its built-in analysis software Analyze (Perkin Elmer) were used to analyze the quantification results of subcutaneous and visceral fat in mice. The obtained fat volume was normalized by the ratio of fat volume to mouse weight, namely the fat volume index = fat volume (mm^3^)/body weight (g).

### Oil red O staining

The improved Oil Red O staining kit (Catalog number: G1263) was purchased from Solarbio (Beijing Solarbio Science & Technology Co., Ltd.). The experimental procedures were conducted according to the instructions of G1263. 200 μl of reagent A (Oil Red O staining buffer) was added to cover the liver frozen Sects. (8 μm) for 5 min. Excess buffer was removed, and then 200 μl of preheated (25 ℃) reagent B (Oil Red O staining solution) was directly added and the sections were stained at room temperature for 15 min. Excess staining solution was removed, and an equal volume of reagent C (Oil Red O differentiation solution) was added for differentiation for 2 min until the sections showed uniform coloration. After a 1-min rinse with distilled water, the sections were stained with reagent D (Mayer's hematoxylin staining solution) for 2 min. Following a 1-min rinse with distilled water, the sections were counterstained with tap water for 8 min until the nucleus appeared as clear blue. The slides were mounted using preheated (58 ℃) melted glycerol gelatin sealing agent (Solarbio, S2150). The positive area of Oil Red O staining was quantified to assess the degree of liver steatosis. Liver Oil Red O staining was performed using a NanoZoomer-SQ digital slide scanner, and Image-Pro Plus 6.0 software was used for image analysis.

### Detection of cholesterol content in liver

The liver tissue was homogenized by combining 1 g of tissue with 10 mL of extraction buffer. The homogenate underwent centrifuged at 10,000 g for 10 min at 4 °C, and the supernatant was collected. Cholesterol content was determined using a total cholesterol detection kit (ZCi Bio Co., Ltd. Lot No. ZC-S0412). In a 96-well plate, 20 µL of standard, sample, and extraction blank control were added separately. Subsequently, 180 µL of working solution was added to each well. The mixture was incubated at 37 °C for 15 min, followed by the measurement of absorbance A at 500 nm. The concentration was determined using the formula: TC content (μmol/g fresh weight) = x (sample concentration) ÷ W (mass of the tested sample).

### Hematoxylin–eosin (HE) staining

The fat samples were fixed in 10% neutral buffered formalin for approximately 18 h. After fixation, the samples were washed, dehydrated, and routinely embedded in paraffin. The embedded tissues were cut into 4 μm thick sections, which were then baked at 56 ℃. The sections were stained using the Sakura DRS2000 staining machine and examined under a microscope after neutral adhesive sealing. Fat samples were collected using the NanoZoomer-SQ digital slide scanner for HE staining. Adiposoft software package, loaded with ImageJ (Fiji, version 1.53t), was utilized to measure the size of adipocytes.

### Immunofluorescence

Frozen Sects. (8 μm) of liver tissue were incubated with 5% bovine serum albumin (BSA) for blocking for 15 min. Primary antibodies of CXCL1 (1:500 dilution, Thermo, MA5-23811) was added to the sections and incubated overnight at 4 °C. The sections were washed 3 times with PBS and incubated with goat anti-mouse IgG (1:500 dilution, Invitrogen, A-11001) at 37 °C for 1 h. A ZEISS laser scanning confocal microscopy (Zena, Dogern, Germany) was used to analyze the sections.

### Aging-associated β-galactosidase staining

Frozen Sects. (8 μm) of liver tissue were fixed and washed at room temperature and then were incubated at 37 °C overnight under light-proof conditions with appropriate amounts of pre-configured staining working solution. Slides were sealed by dehydration and were observed under light microscope.

### qRT-PCR

The gene expression was assessed by qRT-PCR. The primer sequences used are shown as follow (Table 1).

After homogenizing the frozen tissue using Tissuelyser24 (Shanghai Jingxin), total RNA was extracted using the RNeasy Micro Kit (QIAGEN, 74004). According to the instruction manual, the total RNA was reverse transcribed into cDNA using the GoScript™ Reverse Transcription System (Promega, A5001). The relative expression level of the gene was detected on a LightCycler 96 system (Roche) using the GoTaq qPCR Master Mix (Promega, A6002), following the instructions provided with the kit. The target mRNA levels were normalized to Actb mRNA levels, and statistical analysis of mRNA levels was conducted using the 2^−ΔΔCt^ method.

### RNA sequencing and bioinformatic analysis

LC-BIO Technology Co., Ltd. in Hangzhou, China conducted RNA sequencing (RNA-seq) on kidney perirenal adipose tissue and liver tissue from C57BL/6 J mice in the Young, Aged, and BZBS groups. In brief, samples were processed using TRIzol (Thermo Fisher, 15596018) to isolate and purify total RNA, which was then evaluated for quantity, purity, and integrity using NanoDrop ND-1000 (NanoDrop, Wilmington, DE, USA) and Agilent Bioanalyzer 2100 (Agilent, CA, USA). Samples meeting specific criteria (concentration > 50 ng/μL, RNA integrity number > 7.0, total RNA > 1 μg) underwent mRNA purification using oligo(dT) magnetic beads, followed by fragmentation, reverse transcription into cDNA, and double-strand synthesis. Size selection, ligation to adapters, PCR amplification, and Illumina NovaSeqTM 6000 sequencing were subsequently performed to generate strand-specific libraries with fragment sizes of approximately 300 bp ± 50 bp. Bioinformatic analysis was performed as previously described [42].

### Statistical analysis

All statistical analyses were performed using GraphPad Prism (version 8.0). Prior to selecting appropriate statistical tests, normal distribution of data (Shapiro–Wilk test) and homogeneity of variances (Levene's test for equal variances) were checked. One-way analysis of variance (ANOVA) was used to assess significant differences in normally distributed data. Kruskal–Wallis test was used for non-normally distributed data analysis. In all analyses, a p-value less than 0.05 was considered statistically significant. All data are presented as mean ± standard deviation (SD).

## Results

### Energy metabolism trajectory across the mice lifespan

**Fig. 1:**
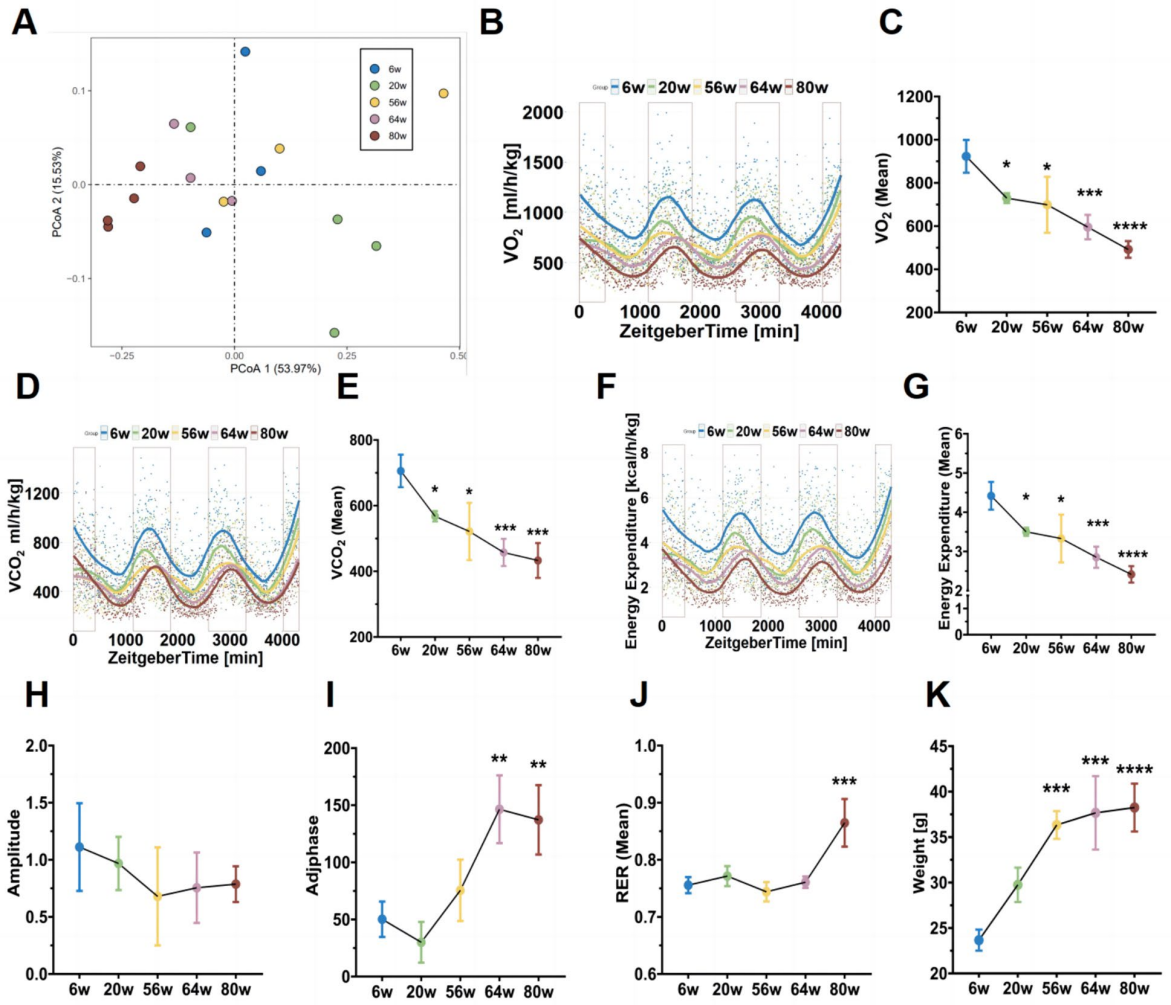
Changes in energy metabolism parameters in mice of different age groups. **A** PCoA plot of differences in energy metabolism in mice of different age groups measured by indirect calorimetry. Each point represents a mouse. Fitted curves and statistical analysis of VO_2_ (**B**, **C**), VCO_2_ (**D**, **E**), and EE (**F**, **G**) in mice of different age groups. Amplitude values of EE (**H**), phase adjustment values of EE (**I**), RER (**J**), and body weight (**K**) in mice of different age groups. n = 3–4. Data are presented as mean ± standard deviation. * indicates significant differences compared to the 6w data. *p < 0.05, **p < 0.01, ***p < 0.001

To depict the trajectory of metabolic changes throughout the lifespan of mice, we conducted indirect calorimetry analysis on five groups of mice of varying ages (Additional file 3: Table S1). Principal coordinate analysis (PCoA) intuitively displayed the differences in metabolic patterns between young and old mice (Fig. 1A). Among the three parameters, oxygen consumption (VO_2_), carbon dioxide production (VCO_2_), and daily energy expenditure (EE), all exhibited similar fluctuation trends, peaking at 6 weeks of age and decreasing over time (Fig. 1B–G). Compared to the 6-week-old mice, energy expenditure decreased by nearly 50% in 80-week-old mice (Fig. 1G). It is worth noting that, while the optimal diurnal cycle of daily energy expenditure remained consistent, the amplitude and adjusted phase values of daily energy expenditure changed (Fig. 1H, I), indicating a gradual disruption of metabolic rhythms during aging. In contrast to the decreasing trends of VO_2_, VCO_2_, and EE, respiratory exchange ratio (RER) showed an increasing trend, particularly after 64 weeks of age (Fig. 1J). RER reflects the ratio of VCO_2_ production to VO_2_ consumption, reflecting energy production efficiency and the main sources of energy substrates. Complete oxidation of glucose molecules requires a specific amount of oxygen and produces an equal amount of carbon dioxide. Therefore, if mammals rely solely on glucose as an energy source, their RER will be 1. Conversely, if fatty acids are used as an energy source, the chemical reactions involved in oxidation require more oxygen, resulting in a decrease in RER to around 0.7. However, compared to equal masses of carbohydrates and proteins, 1 g of fatty acids provides approximately twice the amount of energy [43]. An increase in RER could have two possible explanations: one is related to enhanced glucose metabolism, and the other is related to impaired lipid metabolism. Given the increased body weight (Fig. 1K) and decreased EE in aging mice, it can be inferred that aging is more likely to induce lipid metabolism disorders.

### BZBS mitigates the accumulation of visceral fat and the decline in total energy expenditure caused by aging

**Fig. 2:**
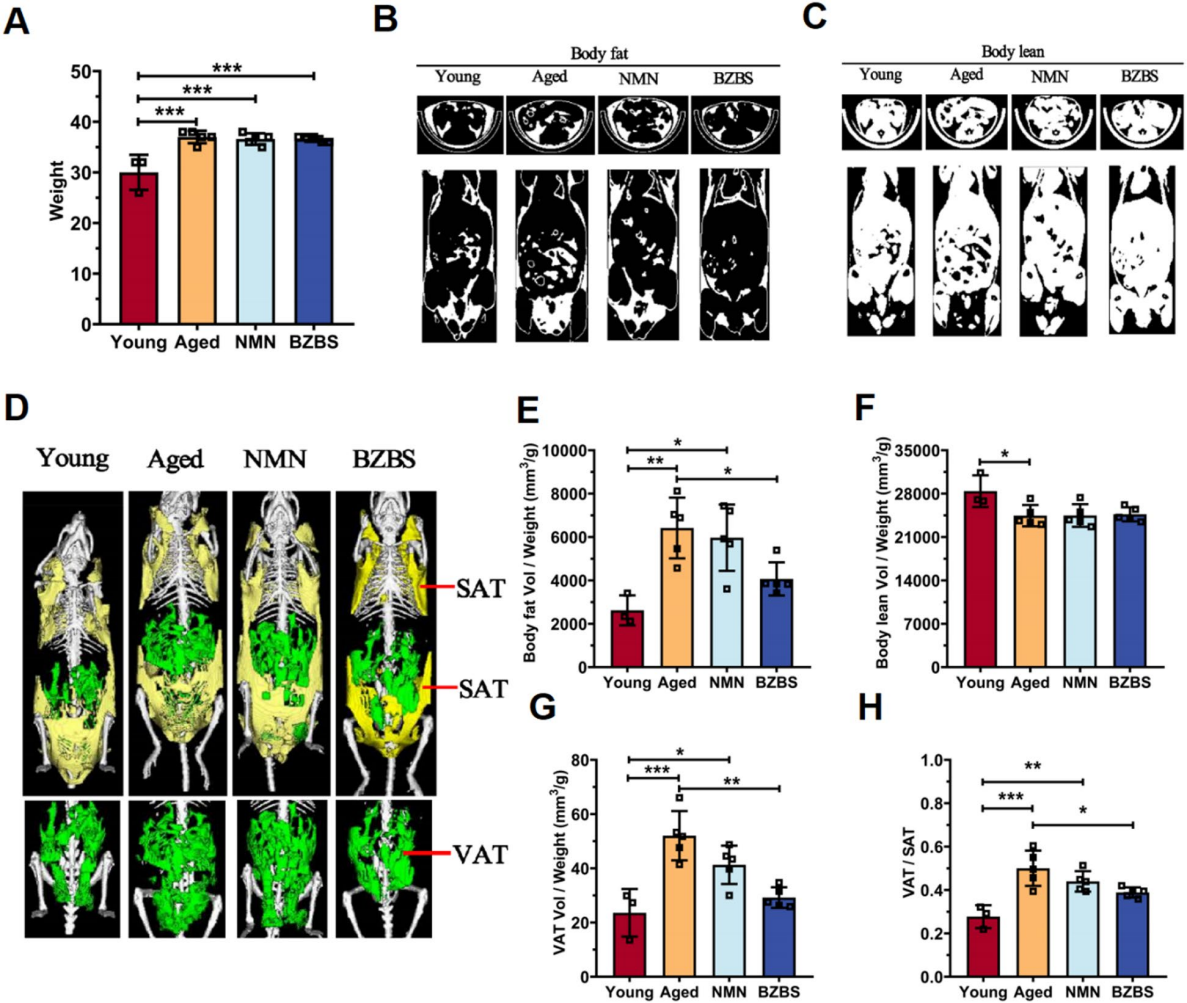
BZBS alleviates age-related visceral fat accumulation. **A** Body weight of young, aged, BZBS-treated, and NMN-treated mice. Micro-CT detection of total fat content (**B**) and lean content (**C**) in mice of each group, bone density change was not measured. **D** Further differentiation of total fat volume into subcutaneous adipose tissue (SAT) and abdominal visceral fat (VAT). Quantification of body fat volume (mm^3^)/body weight (g) (**E**), lean volume (mm^3^)/body weight (g) (**F**), VAT volume (mm^3^)/body weight (g) (**G**), and VAT/SAT ratio (**H**) by Micro-CT. n = 3–5. Data are presented as mean ± standard deviation. *p < 0.05, **p < 0.01, ***p < 0.001

In order to further confirm the lipid metabolism disorder caused by aging, we analyzed the body composition of mice using micro-CT. As shown in Fig. 2A, the overall body weight of aged mice was significantly higher compared to young mice. The increase in body weight observed in aged mice was primarily attributed to the accumulation of fat, rather than a reduction in lean body weight (Fig. 2B–F). Additionally, we examined the body composition of mice administered with NMN (a well-known longevity and metabolic regulation supplement) and BZBS to assess their potential beneficial effects on age-related body composition. BZBS administration significantly reduced fat accumulation in aged mice, whereas NMN treatment had a slight impact on reducing fat content (Fig. 2B–F). By utilizing micro-CT analysis, we were able to acquire the three-dimensional structure and distinguish between subcutaneous and visceral fat in mice. The results showed that both NMN and BZBS administration reduced the relative content of visceral fat (Fig. 2G, H). Especially, BZBS administration significantly decreased the absolute amount of visceral fat, which is closely associated with age-related metabolic disorders such as hypertension, high cholesterol, and insulin resistance [44, 45].

**Fig. 3:**
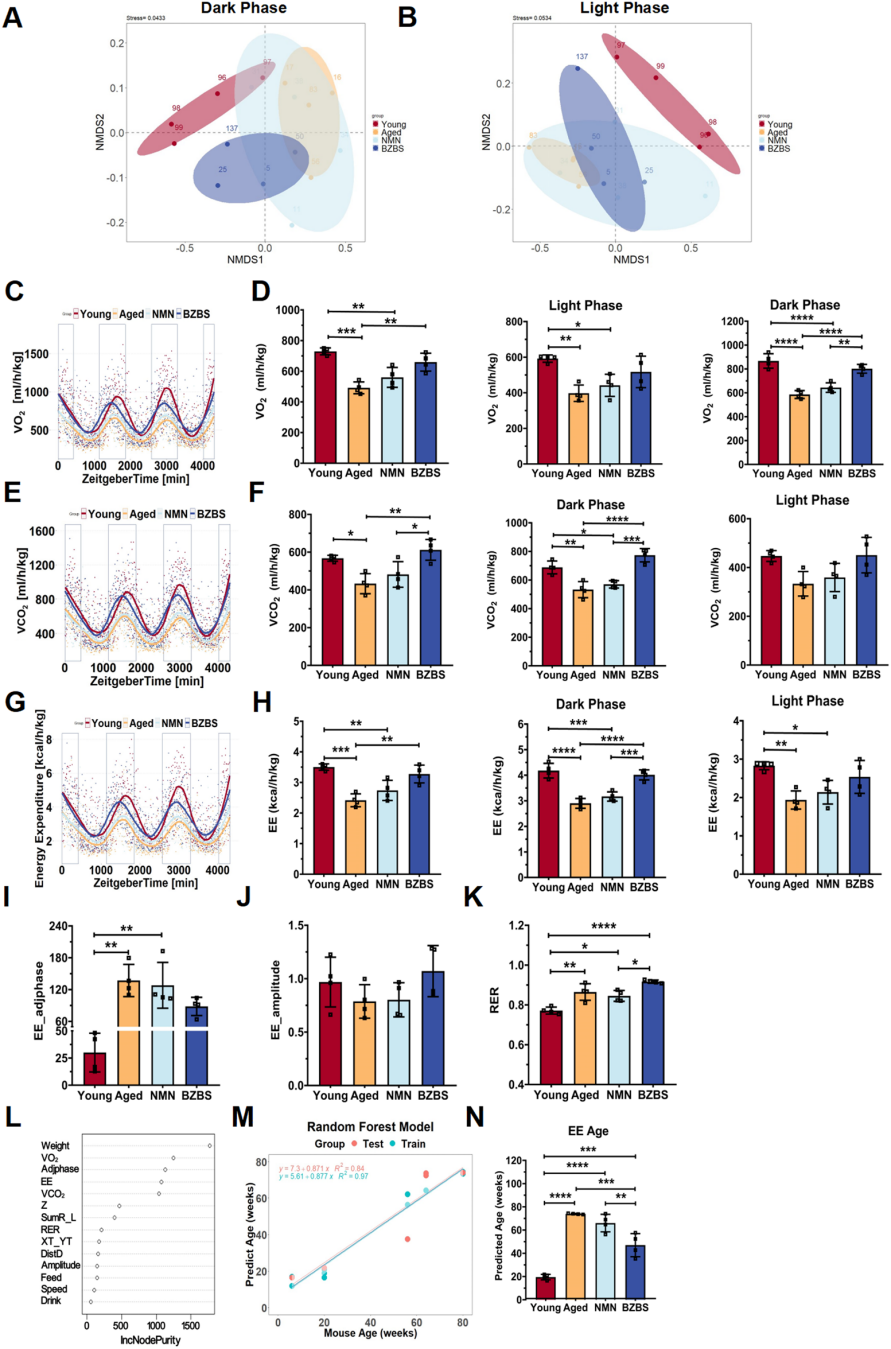
BZBS rejuvenates energy metabolism in aged mice. NMDS plots of dark phase (**A**) and light phase (**B**) showing differences in energy metabolism in young, aged, BZBS-treated, and NMN-treated mice measured by indirect calorimetry. Fitted curves and statistical analysis of VO_2_ (**C**, **D**), VCO_2_ (**E**, **F**), and EE (**G**, **H**) in mice of each group. Amplitude values of EE (**I**), phase adjustment values of EE (**J**), and RER (**K**) in mice of each group. Random forest mathematical models constructed using energy metabolism parameters of mice of different age groups (**L**, **M**), and use of this mathematical model to predict the biological age of EE in young, aged, BZBS-treated, and NMN-treated mice (**N**). n = 4. Statistical significance test was performed using one-way ANOVA and Sidak multiple comparison test. Data are presented as mean ± standard deviation. *p < 0.05, **p < 0.01, ***p < 0.001

Consistent with the results in Fig. 1A, non-metric multidimensional scaling (NMDS) analysis of energy metabolism parameters indicated significant differences in metabolic patterns between young (20-week-old) and aged (80-week-old) mice, especially during the active period (dark phase); and BZBS administration significantly altered the metabolic pattern (Fig. 3A, B). Further data analysis showed that BZBS increased VO_2_, VCO_2_, and EE, especially during the dark phase, without significant effects on the RER (Fig. 3C–K). In order to quantify the age-related changes in energy metabolism and its correlation with actual age, this study constructed a random forest (RF) regression model of energy metabolism parameters using mice of different age groups (see Materials and Methods). The RF algorithm possesses unique advantages in handling small sample sizes and complex data structures [46, 47]. Six most important parameters (Weight, VO_2_, Adjphase, EE, VCO_2_, Z) were determined from the comprehensive random forest model. These parameters were used to develop a more accurate sparse random forest model for predicting the energy expenditure age (EE age) from each mouse (Fig. 3L–N). Surprisingly, mice treated with BZBS exhibited an EE age of 47.032 ± 9.969 weeks, which was approximately 27 weeks younger than that of control aged mice (73.941 ± 0.443).

### BZBS alleviates inflammaging and SASP

**Fig. 4:**
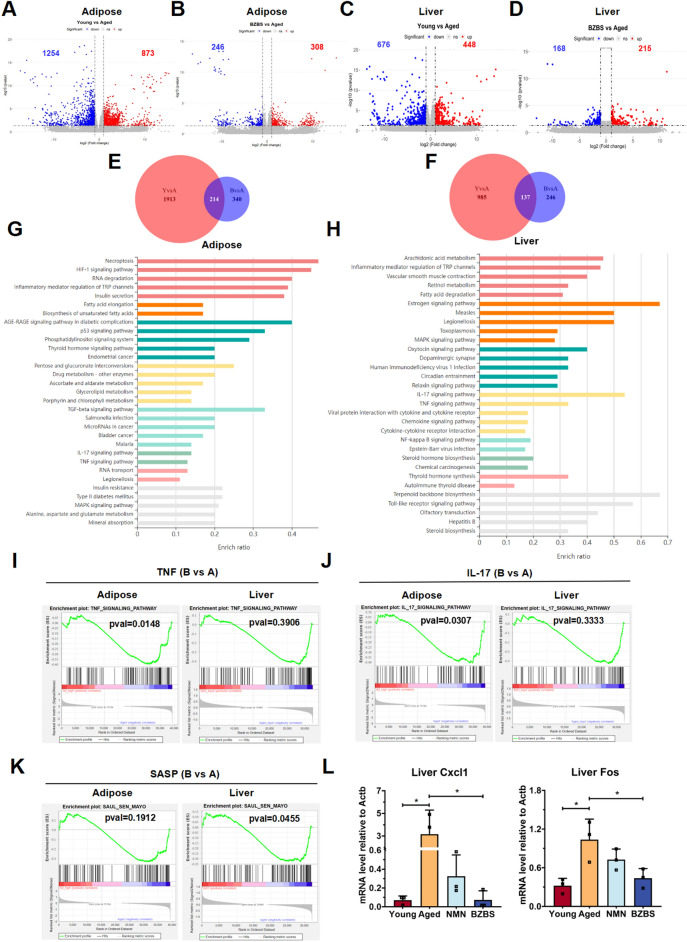
Transcriptome analysis reveals BZBS improving inflammatory and SASP. The number of differentially expressed genes (DEGs) in RNA-seq analysis of young vs. aged and BZBS vs. aged in perirenal fat (**A**, **B**) and liver (**C**, **D**) shown in volcano plots. Intersection of DEGs in Venn diagrams for young vs. aged (Y vs A) and BZBS vs. aged (**B** vs **A**) in perirenal fat (**E**) and liver (**F**). KEGG pathway analysis of intersection DEGs in perirenal fat (**G**) and liver (**H**). Gene Set Enrichment Analysis (GSEA) of TNF signaling pathway (**I**), IL-17 signaling pathway (**J**), and SASP (**K**) in perirenal fat and liver RNA-seq. The mRNA expression of inflammation-related genes by qRT-PCR (**L**)

In order to further explore the mechanism of BZBS in delaying metabolic aging, we performed transcriptome sequencing on lipid metabolism-related tissues (perirenal adipose tissue samples and liver tissue samples) of three groups of mice (the young group, the old group, and the BZBS-treated group). Overall, an average of 39,270 genes was obtained for each sample. For perirenal adipose tissue (prWAT), 2,127 differentially expressed genes (DEGs), including 873 upregulated genes and 1,254 downregulated genes, were identified in the “Young vs. Aged” comparison group (Y vs A) (Fig. 4A). In the “BZBS vs. Aged” comparison group (B vs A), 554 DEGs, including 308 upregulated genes and 246 downregulated genes, were identified (Fig. 4B). Similarly, for the liver, there were 448 upregulated DEGs and 676 downregulated DEGs in the “Young vs. Aged” group (Fig. 4C). In the “BZBS vs. Aged” comparison group, 215 upregulated DEGs and 168 downregulated DEGs were identified (Fig. 4D). For perirenal adipose tissue, there were 214 common genes between the “Y vs A” comparison group and the “B vs A” comparison group (Fig. 4E). Similarly, in the liver, there were 137 common genes between the “Y vs A” comparison group and the “B vs A” comparison group (Fig. 4F). The Kyoto Encyclopedia of Genes and Genomes (KEGG) enrichment analysis of the common genes in perirenal adipose tissue and liver indicated that the main pathways affected by BZBS intervention were related to inflammation, including necroptosis, TGFβ pathway, TNF signaling pathway, IL-17 signaling pathway, NF-κB pathway, and inflammation mediator regulation of TRP channels (Fig. 4G, H). The gene set enrichment analysis (GSEA) analysis further confirmed the upregulation of TNF and IL-17 signaling pathways in aging mouse tissues. However, BZBS could alleviate this inflammatory response and reduce SASP expression (Fig. 4I–K). Among the DEGs regulated by BZBS in visceral adipose tissue and liver, 17 genes were found to be co-regulated (Additional file 1: Figure S1A, B). qRT-PCR confirmed the downregulation of two inflammation-related genes, *Cxcl1* and *Fos*, by BZBS (Fig. 4L). Immunofluorescence analysis confirmed a significant elevation in Cxcl1 protein levels in the livers of aged mice, whereas BZBS treatment led to a reduction in Cxcl1 protein expression (Additional file 1: Figure S1C, D). Accordingly, the activity of liver β-galactosidase in the aging group after BZBS treatment was reduced (Additional file 1: Figure S1E). To further explore the impact of BZBS on age-related cardiovascular diseases and metabolic risk factors, we employed the DisGeNET platform (https://www.disgenet.org/) to conduct an analysis of gene-disease relationships (Additional file 2: Figure S2A). The results indicated that BZBS can prevent aging-related diseases such as obesity, diabetes, hypertension, and atherosclerosis by reducing chronic inflammation-related genes.

### BZBS enhances lipid metabolism in the liver of naturally aging mice

**Fig. 5:**
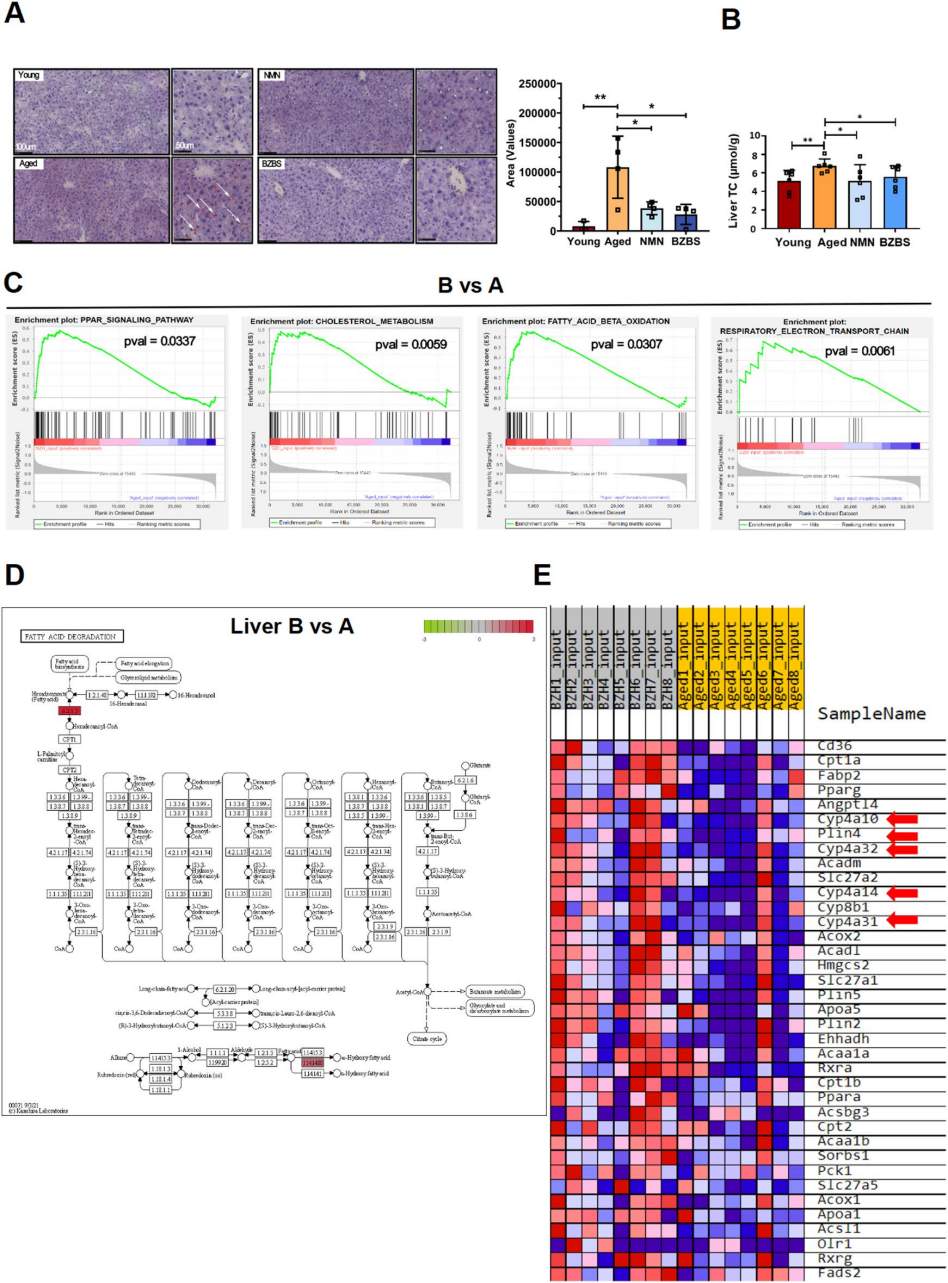
BZBS improves lipid metabolism related to aging in the liver. **A** Comparison of oil red O staining and quantification of positive staining area in the livers. **B** Hepatic cholesterol of each group. **C** GSEA of DEGs (differentially expressed genes) in BZBS vs. Aged (**B** vs. **A**) through liver RNA-seq. **D** KEGG pathway map of DEGs involved in fatty acid degradation. **E** Heatmap showing the expression of PPAR pathway genes in the liver of BZBS mice and aged mice. Data is presented as mean ± standard deviation. *p < 0.05, **p < 0.01

The liver plays a crucial role in lipid metabolism. Fatty acids in the liver can undergo β-oxidation, a process that generates energy. These fatty acids can be stored as triglycerides within lipid droplets or packaged into very low-density lipoproteins (VLDL) and released into the bloodstream [48]. Oil red O staining of the liver revealed a significant increase in the positive staining area in aged mice compared to young mice (Fig. 5A). Both BZBS and NMN treatment significantly reduced the positive staining area. Consistent with the Oil red O staining result, we detected significantly elevated cholesterol levels in the livers of aging mice, while BZBS treatment alleviated the age-related accumulation of hepatic cholesterol (Fig. 5B). The liver GSEA analysis of liver transcriptome data revealed that BZBS upregulated genes involved in the PPAR signaling pathway, cholesterol metabolism, fatty acid β-oxidation, and respiratory electron transport chain (Fig. 5C). Among them, BZBS upregulated the *Acsl6* gene, which encodes a key rate-limiting enzyme (EC:6.2.1.3) for fatty acid β-oxidation (Fig. 5D). In the BZBS-activated PPAR signaling pathway, genes such as *Cyp4a32*, *Cyp4a31*, *Cyp4a14*, *Cyp4a10*, and *Plin4* were upregulated (Fig. 5E), and these genes are involved in the regulation of glucose and lipid metabolism in db/db diabetic mice [49].

**Fig. 6:**
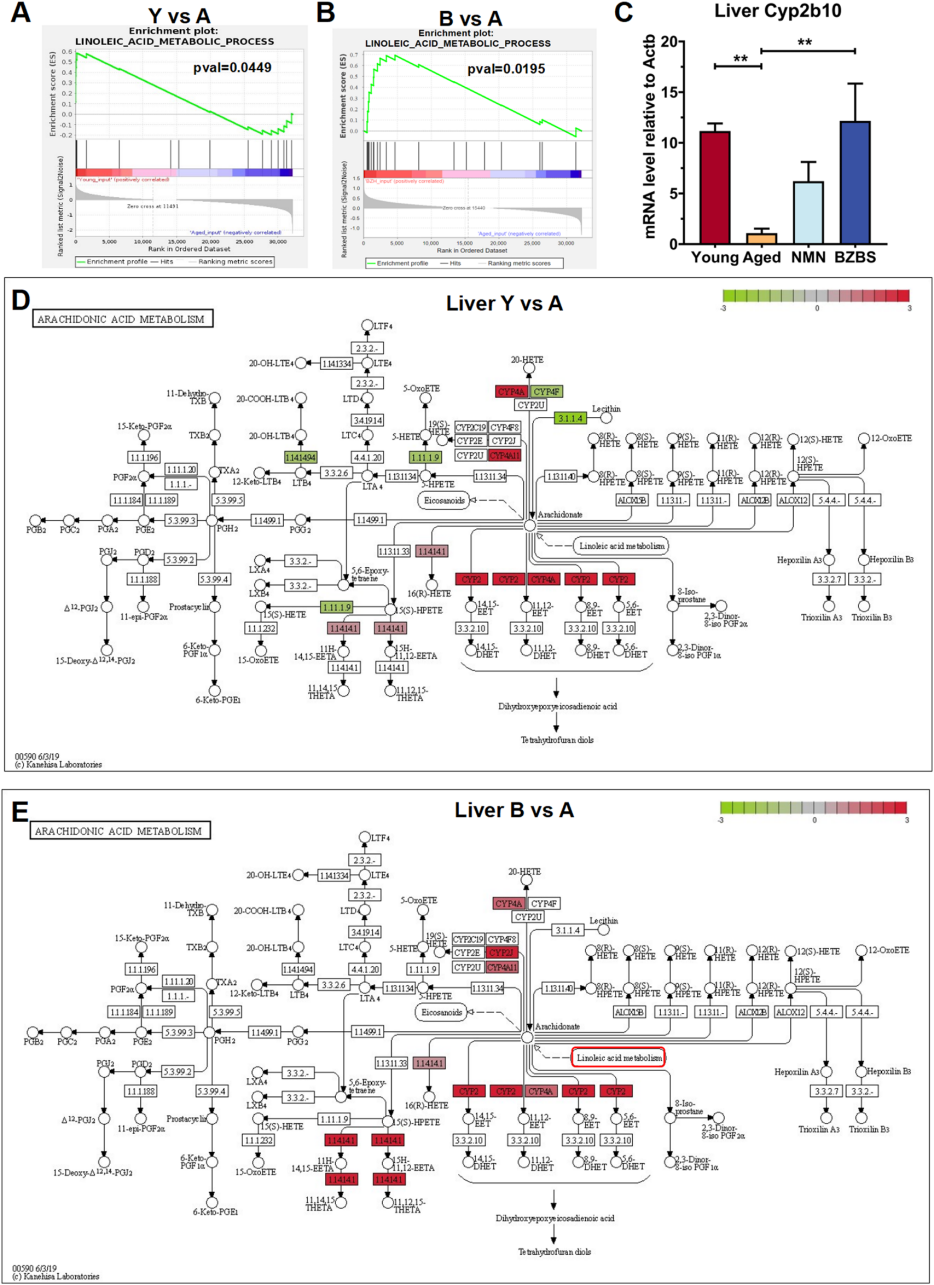
Administration of BZBS enhances the metabolism of linoleic acid (LA) and arachidonic acid (AA) in the liver. GSEA of DEGs (differentially expressed genes) for Y vs A (**A**) and B vs A (**B**) in liver RNA-seq analysis is conducted. **C** The relative mRNA levels of *Cyp2b10* are determined by qRT-PCR (n = 3). KEGG pathway maps of AA metabolism DEGs for Y vs A **D** and B vs A **E** in liver RNA-seq analysis. Data is presented as mean ± standard deviation. *p < 0.05, **p < 0.01

In the investigation of lipid metabolism imbalance induced by aging, we observed a reduction in the metabolism of linoleic acid (LA) within the liver tissue of aged mice. However, treatment with BZBS reversed this trend and potentiated LA metabolism (Fig. 6A, B). LA, as an essential fatty acid, is involved in the metabolism of arachidonic acid (AA) and is associated with various physiological and pathological conditions. The intake of LA is related to the reduction of cardiovascular disease and diabetes risk [50–52]. Further analysis of mouse liver sequencing data revealed that genes encoding proteases involved in the metabolism of arachidonate to 15-hydroxy-11,12-epoxyeicosatrienoic acid (15-H-11,12-HEETA), 11,12,15-trihydroxyeicosatrienoic acid (11,12,15-THETA), and epoxyeicosatrienoic acids (EETs) were downregulated in aged mice (Fig. 6C, D). Hydroxy epoxyeicosatrienoic acid (HEETA) and trihydroxyeicosatrienoic acids (THETA) are endothelium-derived hyperpolarizing factors that induce vascular dilation [53]. EETs have a protective effect on maintaining vascular homeostasis and anti-inflammatory effects. They have also been linked to cardiovascular diseases and metabolic disorders related to obesity [54–57]. The upregulation of cytochrome P450 (CYP) enzyme genes, such as Cyp2b10, by BZBS may enhance the metabolism of arachidonate to EETs (5,6-EET, 8,9-EET, 11,12-EET, and 14,15-EET), leading to a decrease in the production of pro-inflammatory substance prostaglandin E2 (PGE2) (Fig. 6C, E). These results indicate that the positive regulation of linoleic acid by BZBS is a potential mechanism for its alleviation of tissue inflammation.

### BZBS enhances glycolysis and thermogenesis in the visceral adipose tissue of naturally aged mice

**Fig. 7:**
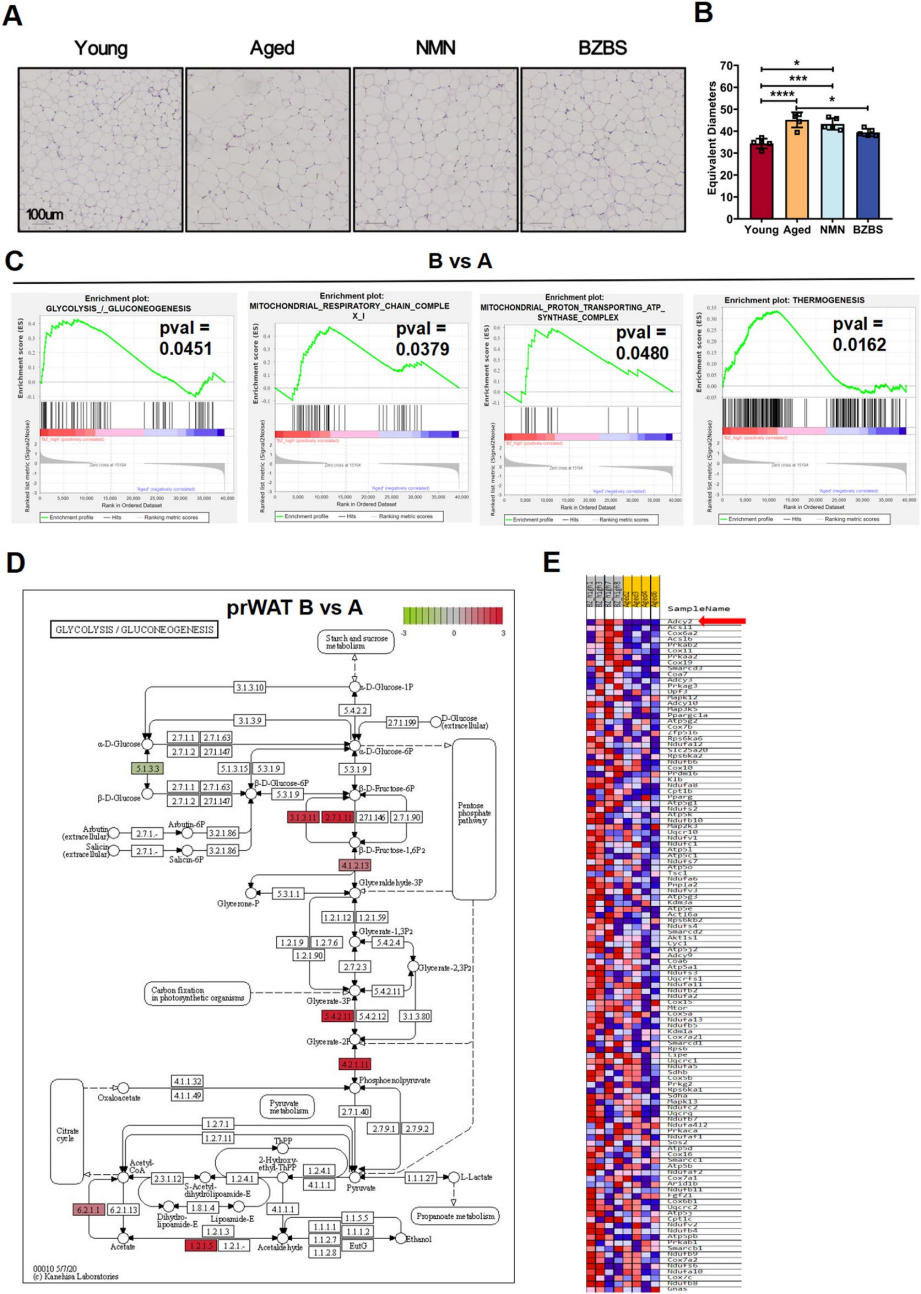
BZBS enhances thermogenesis and glucose metabolism in adipose tissue. Comparison of HE staining of adipose tissue **A** and quantitative comparison of the equivalent diameter of adipocytes (**B**). **C** GSEA of DEGs in **B** vs **A** in RNA-seq of adipose tissue. **D** KEGG pathway diagram of glycolysis/gluconeogenesis DEGs. **E** Heatmap showing the expression of thermogenesis pathway genes in BZBS mice and aged mice

Adipose tissue is one of the main organs responsible for fat storage and energy metabolism. The energy metabolism of thermogenic adipocytes depends on glucose and fatty acids, with enhanced glycolysis and fatty acid oxidation contributing to uncoupled respiration and heat production [58]. HE staining of visceral adipose tissue from aged mice revealed hypertrophy of adipocytes, which was reversed upon administration of BZBS (Fig. 7A, B). Interestingly, GSEA analysis of transcriptomic data from perirenal fat indicated that, compared to lipid metabolism, BZBS had a more significant upregulating effect on glycolysis/gluconeogenesis pathway. Furthermore, there was a significant upregulation of mitochondrial respiratory chain complex I, mitochondrial proton-translocating ATP synthase complex, and thermogenesis pathway after BZBS treatment (Fig. 7C). Within the glycolysis pathway, BZBS treatment significantly increased the levels of phosphofructokinase (EC:2.7.1.11), as well as enolase (EC:4.2.1.11), which are key enzymes involved in the conversion of β-D-Fructose-6P to phosphoenolpyruvate (Fig. 7D). In the thermogenesis pathway, BZBS significantly upregulated the expression of *Adcy2* gene (Fig. 7E). Our findings demonstrate that BZBS improves the metabolism and thermogenic function of visceral adipose tissue in aging mice, thus elucidating the mechanism behind BZBS-induced increase in energy expenditure.

## Discussion

Natural aging in mice results in metabolic dysregulation, manifested as a progressive decline in EE with age and a change in substrate utilization from fat to glucose. This study utilized a random forest mathematical model to construct the EEAI and applied it to assess the capability of BZBS in counteracting age-related decline in EE. Micro-CT and pathological examinations verified the beneficial effects of BZBS on age-related visceral fat accumulation and hepatic steatosis. Transcriptomic sequencing results demonstrated the significant improvement of BZBS in age-induced imbalance of glucose and lipid metabolism and mitochondrial dysfunction through regulation of SASP-mediated inflammatory responses.

Metabolic imbalances, driven by internal and external environmental shifts during aging, are considered key players in the biology of senescence [5, 61, 62]. To unravel this link, we tracked the energy expenditure of C57BL/6 J mice from 6 to 80 weeks of age, charting their metabolic journey. Our analysis revealed a decline in energy metabolism with age, alongside a dampening of its flexibility, particularly manifested in disrupted diurnal rhythms and impaired substrate utilization (Fig. 1). Notably, older mice exhibited a higher respiratory exchange ratio (RER, ~ 0.85) compared to their prime (~ 0.75), hinting at a shift in fuel preference during aging (Fig. 1J). Previous studies implicate impaired lipid mobilization and increased non-adipocyte lipid synthesis in age-related fat accumulation in organs like the liver, adipose tissue, and vasculature [63–65]. Micro-CT and histopathological staining confirmed this lipid deposition in the visceral fat and liver of aged mice (Figs. 2, 5A, 7A).

Recent advancements have seen the development of various biomarkers for predicting biological age, including DNA methylation clocks and inflammatory clocks [59, 60]. In our prior research, we employed the well-established liver DNA methylation clock to estimate biological age of the mice treated by BZBS. Our observations indicated that BZBS reverses the biological aging process in mice [40]. Building on this, we developed the first energy expenditure-based aging index using metabolic cage parameters. This allowed us to evaluate age-related alterations in metabolic flexibility and biological age, bridging the gap between metabolic shifts and aging prediction. By comparing the results of the liver DNA methylation clock and the EEAI clock, we found that both clocks accurately reflect the actual age of normal mice [40]. Moreover, BZBS consistently reduced the biological age of mice by approximately 50% according to both clocks, partially validating the EEAI clock's accuracy. Additionally, the EEAI clock, a computational model anchored in fundamental metabolic parameters, offers the benefit of non-invasive monitoring over traditional methods like DNA methylation and blood clocks, enabling longitudinal monitoring of animals. Our findings reveal that BZBS treatment maintains the diurnal rhythm of energy metabolism and effectively revitalizes the biological aging of energy expenditure (Fig. 3).

Among the three main types of mammalian adipocytes—brown (responsible for heat production) and beige (with similar function) versus white (responsible for fat storage)—visceral fat accumulates to a greater volume than subcutaneous fat, which can increase chronic inflammation risk, impair organ function, and accelerate aging, further contributing to the development of various age-related diseases like heart disease, diabetes, and even cancers [58, 66–69]. In our study, we focused on perirenal fat for RNA sequencing analysis due to its crucial role in modulating kidney function through the secretion of cytokines like leptin and adiponectin, as well as its significant contribution to the aging process. Transcriptome analysis of perirenal fat confirms that BZBS administration enhances thermogenesis and glycolysis (Fig. 7). The liver, a central player in lipid metabolism, also exhibits age-induced metabolic aberrations [48, 70, 71]. Hepatic transcriptome analysis revealed downregulated long chain fatty acid transporter activity, fatty acid degradation, and fatty acid β-oxidation in aged mice. Notably, BZBS administration not only boosted fatty acid degradation and β-oxidation but also enhanced triglyceride catabolic process, cholesterol metabolism and mitochondrial function (Fig. 5). The PPAR signaling pathway, a master regulator of fatty acid β-oxidation [72], was significantly upregulated in BZBS-treated mice, as evidenced by increased expression of *Cyp4a32*, *Cyp4a31*, *Cyp4a14*, *Cyp4a10*, and *Plin4* genes.

Recent works by Baker and Rutter highlight the role of metabolites as signaling molecules that influence processes such as nutrient sensing, cell differentiation, immune response, and cytokine secretion [73]. LA and AA metabolism generates various bioactive metabolites, including prostanoids, leukotrienes, and lipoxins, but also extends to vasodilatory and anti-inflammatory metabolites such as hydroxyeicosatetraenoic acids (HEETAs), trioxilin hydroxyeicosatetraenoic acids (THETAs), and epoxyeicosatrienoic acids (EETs) [52, 54–56]. Our transcriptome analysis in aged mouse liver revealed a downregulation of arachidonate metabolism towards 15-H-11,12-HEETA, 11,12,15-THETA, and EETs, which was reversed by BZBS treatment (Fig. 6). This finding aligns with metabolomic data from Huang et al., showing that BZBS increased EET levels in the serum of high-fat diet-induced atherosclerosis Ovx/ApoE ^−/−^ mice [33]. Collectively, these findings support the notion that metabolic dysfunction fuels chronic inflammation, and importantly, suggest that BZBS treatment may mitigate this inflammatory state.

Notably, similar to the results from liver transcriptome, the adipose tissue transcriptome also displayed marked inflammatory traits. Specifically, both tissues demonstrated enriched IL-17 and TNF signaling pathways (Fig. 4). This suggests that chronic inflammation is a common phenomenon in different organs during the natural aging process. The TNF and IL-17 signaling pathways are involved in regulating metabolism and promoting an excessively inflammatory environment [74, 75]. Moreover, beyond chronic inflammation, we also noticed that a large number of SASP genes were upregulated in both tissues. It is generally believed that the chronic inflammatory microenvironment fosters senescence and reprograms the cellular landscape through SASP, pushing neighboring cells towards apoptosis or senescence. This closed loop fuels a perpetual cycle of inflammation and aging [11, 13]. Nevertheless, BZBS exhibits remarkable efficacy in dampening inflammatory responses and reducing SASP burden. Downregulation of *Cxcl1* and *Fos* genes by BZBS deserves particular attention. Cxcl1 primarily recruits neutrophils through Cxcr2 [11, 76, 77], and while their number may not decline drastically with age, their functionality (phagocytosis, adhesion, chemotaxis) suffers under the chronic inflammatory burden [11]. c-Fos, one of the components of activator protein-1 (AP-1), along with c-Jun, is upregulated by TNF-α, intensifying AP-1 binding to the TGF-β1 promoter and consequently boosting TGF-β1 transcription, thereby promoting fibrosis [78, 79]. Previous research identified Fos as a hypermethylated gene modulated by BZBS, and our transcriptome data corroborates this regulation, suggesting DNA methylation as a potential mechanism for BZBS's anti-inflammatory action [39, 40]. Furthermore, inflammaging serves as a potent risk factor for age-related metabolic diseases like atherosclerosis, hypertension, type 2 diabetes, and non-alcoholic fatty liver disease [60]. Utilizing the DisGeNET resource, we conducted a preliminary exploration of BZBS's potential to regulate aging-related cytokines, paving the way for future investigations into its use in preventing and treating obesity, diabetes, hypertension, atherosclerosis, and other age-related metabolic ailments (Additional file 2: Figure S2).

This study also has some limitations. All experimental subjects are male mice. The number of experimental animals is relatively small, and the methods for investigating the mechanism of BZBS are relatively singular. With the development of systems biology and high-throughput sequencing technologies, the research on TCM formulas is shifting towards a "multi-targets, multi-components" paradigm through the exploration of TCM's role in preventing and treating diseases using a multi-omics approach [80]. Future research should employ multi-omics methods to reveal the mechanism of BZBS in regulating aging and preventing age-related diseases from different perspectives.

## Conclusions

**Fig. 8:**
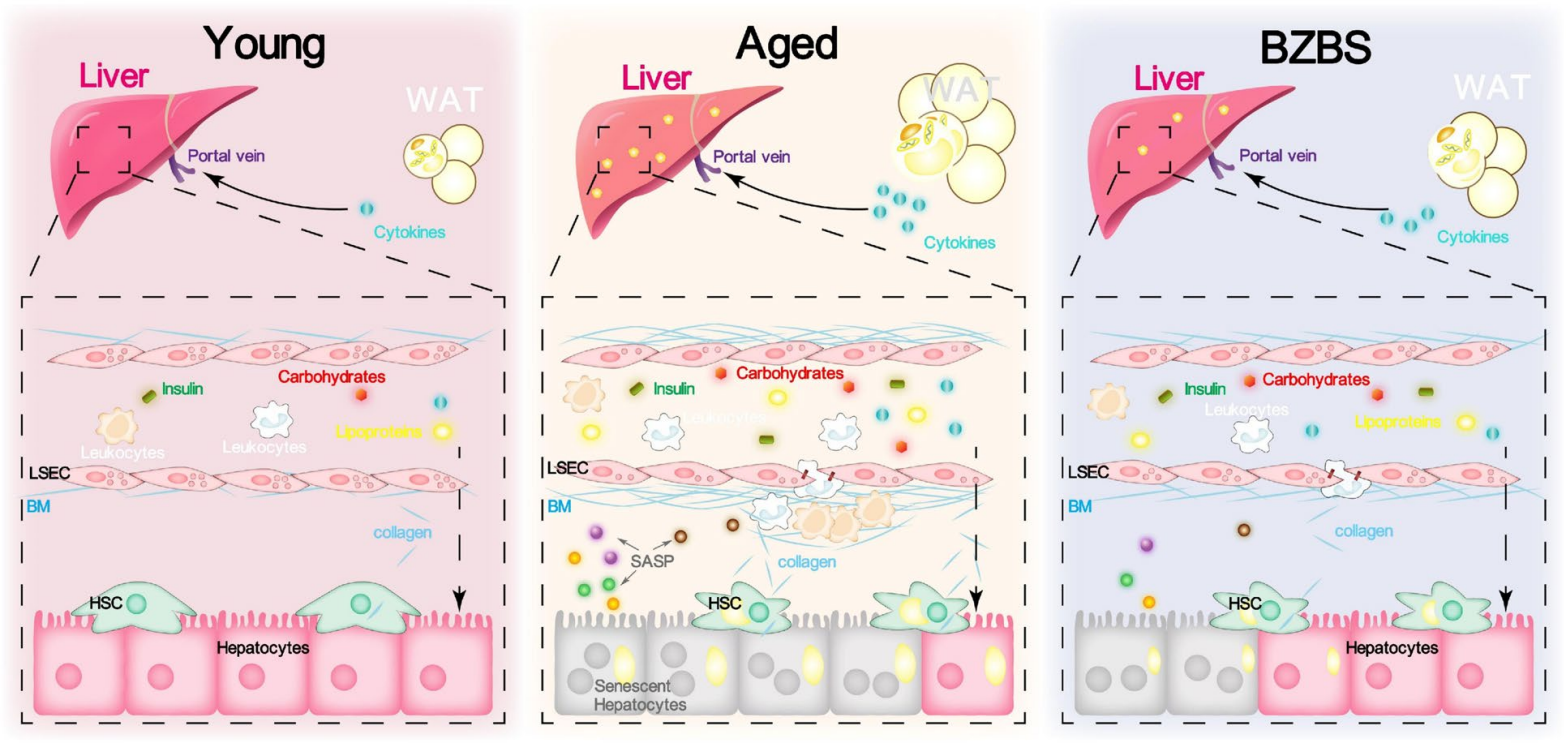
Schematic illustration of the potential mechanism by which BZBS ameliorates age-induced visceral fat accumulation and hepatic steatosis

This study investigated the effects of BZBS on age-related metabolic decline and inflammaging in naturally elderly mice (Fig. 8). Our research findings indicated that natural aging led to decreased EE, shifted substrate utilization, and visceral fat accumulation and hepatic steatosis. BZBS can increase EE, revitalize the diurnal rhythm of energy metabolism, rejuvenated the biological age of EE, and alleviate visceral fat accumulation and hepatic steatosis caused by aging. Mechanistically, BZBS enhanced thermogenesis, glycolysis, fatty acid degradation, triglyceride catabolism, cholesterol metabolism, and mitochondrial function, and alleviated SASP, TNF signaling pathway, and IL-17 signaling pathway. Overall, BZBS demonstrates promising potential in alleviating age-related metabolic decline and inflammaging, paving the way for its potential use in promoting healthy aging and preventing age-related diseases.

### Supplementary Information

**Additional file 1: Figure S1. **BZBS reduces liver senescence cells accumulation in mice. (A) The Venn diagram displays the intersection quantity of DEGs (Differentially Expressed Genes) between perirenal fat and liver BZBS vs. Aged (B vs A). (B) The bar chart shows the expression of intersection inflammation-related genes. (C) The Immunofluorescence of Cxcl1 in the mouse liver. (D) The aging-associated β-galactosidase staining of mouse liver. Data is presented as mean ± standard deviation. *p < 0.05, **p < 0.01.

**Additional file 2: Figure S2. **BZBS reduces susceptibility to age-related metabolic diseases. (A) Correlation analysis of inflammation-related genes in the TNF signaling pathway, IL-17 signaling pathway, and SASP pathway with age-related metabolic diseases in the BZBS and Aged comparison groups.

**Additional file 3: Table S1-S16. **Raw data refer to table.

## Data Availability

The datasets supporting the conclusions of this article are included within the article and its additional files ( Additional file 3: Table S1-S16).

## Abbreviations

IL-17: Interleukin-17
TNF: Tumor necrosis factor
SASP: Senescence associated secretory phenotype
BZBS: Bazi Bushen
EEAI: Energy Expenditure Aging Index
NMN: β-Nicotinamide Mononucleotide
qRT-PCR: Real-time quantitative polymerase chain reaction
PPAR: Peroxisome proliferators-activated receptor
TCM: Traditional Chinese medicine
UPLC/MS: Ultra-performance liquid chromatography/mass spectrometry
VO_2_: Oxygen consumption
VCO_2_: Carbon dioxide production
RER: Respiratory exchange ratio
EE: Energy expenditure
XT: The number of times the X-axis infrared light is interrupted by all activities of the mouse
YT: The number of times the Y-axis infrared light is interrupted by all activities of the mouse
Z: The number of times the Z-axis infrared light is interrupted by all activities of the mouse
DistD: The difference in the movement distance
SumR + L: The sum of the number of left and right turns of the mouse running wheel
PCoA: Principal coordinates analysis
Adjphase: Adjusted phase values of mean daily energy expenditure
Amplitude: Amplitude values of mean daily energy expenditure
SAT: Subcutaneous adipose tissue
VAT: Visceral adipose tissue
HE: Hematoxylin–eosin
*Actb*: Actin, beta
*Cxcl1*: C-X-C motif chemokine ligand 1
*Cyp2b10*: Cytochrome P450, family 2, subfamily b, polypeptide 10
*Fos*: FBJ osteosarcoma oncogene
RNA-seq: RNA-sequencing
ANOVA: One-way analysis of variance
SD: Standard deviation
NMDS: Non-metric multidimensional scaling
RF: Random forest
EE age: Energy expenditure age
prWAT: Perirenal adipose tissue
DEGs: Differentially expressed genes
Y vs A: "Young vs. Aged" comparison group
B vs A: "BZBS vs. Aged" comparison group
KEGG: Kyoto Encyclopedia of Genes and Genomes
TGFβ: Transforming growth factor-beta
NF-κB: Nuclear factor kappa-B
TRP: Transient receptor potential
GSEA: Gene set enrichment analysis
VLDL: Very low-density lipoproteins
*Acsl6*: Acyl-CoA synthetase long chain family member 6
*Cyp4a32*: Cytochrome P450, family 4, subfamily a, polypeptide 32
*Cyp4a31*: Cytochrome P450, family 4, subfamily a, polypeptide 31
*Cyp4a14*: Cytochrome P450, family 4, subfamily a, polypeptide 14
*Cyp4a10*: Cytochrome P450, family 4, subfamily a, polypeptide 10
*Plin4*: Perilipin 4
LA: Linoleic acid
AA: Arachidonic acid
EETs: Epoxyeicosatrienoic acids
HEETA: Hydroxy epoxyeicosatrienoic acid
THETA: Trihydroxyeicosatrienoic acids
CYP: Cytochrome P450
PGE2: Prostaglandin E2
ATP: Adenosine triphosphate
*Adcy2*: Adenylate cyclase 2
Cxcr2: C-X-C Motif Chemokine Receptor 2
AP-1: Activator protein-1

## References

[1] Pontzer H, Yamada Y, Sagayama H, Ainslie PN, Andersen LF, Anderson LJ (2021). Daily energy expenditure through the human life course. Science.

[2] Zoico E, Rubele S, De Caro A, Nori N, Mazzali G, Fantin F (2019). Brown and beige adipose tissue and aging. Front Endocrinol.

[3] Mancuso P, Bouchard B (2019). The impact of aging on adipose function and adipokine synthesis. Front Endocrinol.

[4] Chait A, den Hartigh LJ (2020). Adipose tissue distribution, inflammation and its metabolic consequences, including diabetes and cardiovascular disease. Front Cardiovasc Med.

[5] López-Otín C, Blasco MA, Partridge L, Serrano M, Kroemer G (2023). Hallmarks of aging: an expanding universe. Cell.

[6] Manini TM (2010). Energy expenditure and aging. Ageing Res Rev.

[7] Drożdż K, Nabrdalik K, Hajzler W, Kwiendacz H, Gumprecht J, Lip GYH (2021). Metabolic-associated fatty liver disease (mafld), diabetes, and cardiovascular disease: associations with fructose metabolism and gut microbiota. Nutrients.

[8] Deprince A, Haas JT, Staels B (2020). Dysregulated lipid metabolism links nafld to cardiovascular disease. Mol Metab.

[9] Pirillo A, Casula M, Olmastroni E, Norata GD, Catapano AL (2021). Global epidemiology of dyslipidaemias. Nat Rev Cardiol.

[10] Guo J, Huang X, Dou L, Yan M, Shen T, Tang W (2022). Aging and aging-related diseases: from molecular mechanisms to interventions and treatments. Signal Transduct Target Ther.

[11] Li X, Li C, Zhang W, Wang Y, Qian P, Huang H (2023). Inflammation and aging: signaling pathways and intervention therapies. Signal Transduct Target Ther.

[12] Hotamisligil GS (2006). Inflammation and metabolic disorders. Nature.

[13] Zhu X, Chen Z, Shen W, Huang G, Sedivy JM, Wang H (2021). Inflammation, epigenetics, and metabolism converge to cell senescence and ageing: the regulation and intervention. Signal Transduct Target Ther.

[14] Kumari R, Jat P (2021). Mechanisms of cellular senescence: cell cycle arrest and senescence associated secretory phenotype. Front Cell Dev Biol.

[15] Wiley CD, Campisi J (2021). The metabolic roots of senescence: mechanisms and opportunities for intervention. Nat Metab.

[16] Kalinkovich A, Livshits G (2017). Sarcopenic obesity or obese sarcopenia: a cross talk between age-associated adipose tissue and skeletal muscle inflammation as a main mechanism of the pathogenesis. Ageing Res Rev.

[17] Stahl EC, Delgado ER, Alencastro F, Lopresti ST, Wilkinson PD, Roy N (2020). Inflammation and ectopic fat deposition in the aging murine liver is influenced by ccr2. Am J Pathol.

[18] Ogrodnik M, Miwa S, Tchkonia T, Tiniakos D, Wilson CL, Lahat A (2017). Cellular senescence drives age-dependent hepatic steatosis. Nat Commun.

[19] Chung KW (2021). Advances in understanding of the role of lipid metabolism in aging. Cells.

[20] Hildebrandt X, Ibrahim M, Peltzer N (2023). Cell death and inflammation during obesity: “know my methods, wat(son)”. Cell Death Differ.

[21] Ahmed B, Sultana R, Greene MW (2021). Adipose tissue and insulin resistance in obese. Biomed Pharmacother.

[22] Franceschi C, Garagnani P, Parini P, Giuliani C, Santoro A (2018). Inflammaging: a new immune-metabolic viewpoint for age-related diseases. Nat Rev Endocrinol.

[23] Ferrucci L, Fabbri E (2018). Inflammageing: chronic inflammation in ageing, cardiovascular disease, and frailty. Nat Rev Cardiol.

[24] Chaib S, Tchkonia T, Kirkland JL (2022). Cellular senescence and senolytics: the path to the clinic. Nat Med.

[25] Palmer AK, Xu M, Zhu Y, Pirtskhalava T, Weivoda MM, Hachfeld CM (2019). Targeting senescent cells alleviates obesity-induced metabolic dysfunction. Aging Cell.

[26] Xu M, Pirtskhalava T, Farr JN, Weigand BM, Palmer AK, Weivoda MM (2018). Senolytics improve physical function and increase lifespan in old age. Nat Med.

[27] Mbara KC, Devnarain N, Owira PMO (2022). Potential role of polyphenolic flavonoids as senotherapeutic agents in degenerative diseases and geroprotection. Pharmaceut Med.

[28] Bjørklund G, Shanaida M, Lysiuk R, Butnariu M, Peana M, Sarac I (2022). Natural compounds and products from an anti-aging perspective. Molecules.

[29] Mora I, Arola L, Caimari A, Escoté X, Puiggròs F (2022). Structured long-chain omega-3 fatty acids for improvement of cognitive function during aging. Int J Mol Sci.

[30] Lam YY, Peterson CM, Ravussin E (2013). Resveratrol vs. Calorie restriction: data from rodents to humans. Exp Gerontol.

[31] Bereswill S, Muñoz M, Fischer A, Plickert R, Haag L, Otto B (2010). Anti-inflammatory effects of resveratrol, curcumin and simvastatin in acute small intestinal inflammation. PLoS ONE.

[32] Bisht K, Wagner K, Bulmer AC (2010). Curcumin, resveratrol and flavonoids as anti-inflammatory, cyto- and dna-protective dietary compounds. Toxicology.

[33] Huang D, Hu H, Chang L, Liu S, Liang J, Song Y (2020). Chinese medicine bazi bushen capsule improves lipid metabolism in ovariectomized female apoe-/- mice. Ann Palliat Med.

[34] Yu M, Hung T, Wang C, Wu S, Yang T, Yang C (2021). Neochlorogenic acid attenuates hepatic lipid accumulation and inflammation via regulating mir-34a in vitro. Int J Mol Sci.

[35] Yan Y, Li Q, Shen L, Guo K, Zhou X (2022). Chlorogenic acid improves glucose tolerance, lipid metabolism, inflammation and microbiota composition in diabetic db/db mice. Front Endocrinol.

[36] Zhou Y (2020). The protective effects of cryptochlorogenic acid on β-cells function in diabetes in vivo and vitro via inhibition of ferroptosis. Diabetes Metabol Synd Obes.

[37] Sun B, Zhang R, Liang Z, Fan A, Kang D (2021). Hyperoside attenuates non-alcoholic fatty liver disease through targeting nr4a1 in macrophages. Int Immunopharmacol.

[38] Lei P, Lü J, Yao T, Zhang P, Chai X, Wang Y (2023). Verbascoside exerts an anti-atherosclerotic effect by regulating liver glycerophospholipid metabolism. Food Sci Human Wellness.

[39] Huang D, Wang X, Zhu Y, Gong J, Liang J, Song Y (2021). Bazi bushen capsule alleviates post-menopausal atherosclerosis via gper1-dependent anti-inflammatory and anti-apoptotic effects. Front Pharmacol.

[40] Mao X, Hou Y, Fang C, Ma K, Zhang S, Guo Z (2023). Bazi bushen mitigates epigenetic aging and extends healthspan in naturally aging mice. Biomed Pharmacother.

[41] Yeh C, Shen Z, Wang T, Kao C, Teng Y, Yeh T (2022). Hesperetin promotes longevity and delays aging via activation of cisd2 in naturally aged mice. J Biomed Sci.

[42] Lv J, Hu Y, Li L, He Y, Wang J, Guo N (2023). Targeting fabp4 in elderly mice rejuvenates liver metabolism and ameliorates aging-associated metabolic disorders. Metabolism Clin Exp.

[43] Meng F, Zhu X, Zhao H, Lu F, Lu Y, Lu Z (2021). Improve production of pullulanase of bacillus subtilis in batch and fed-batch cultures. Appl Biochem Biotechnol.

[44] Agrawal S, Wang M, Klarqvist MDR, Smith K, Shin J, Dashti H (2022). Inherited basis of visceral, abdominal subcutaneous and gluteofemoral fat depots. Nat Commun.

[45] Ponti F, Santoro A, Mercatelli D, Gasperini C, Conte M, Martucci M (2019). Aging and imaging assessment of body composition: from fat to facts. Front Endocrinol.

[46] Luan J, Zhang C, Xu B, Xue Y, Ren Y (2020). The predictive performances of random forest models with limited sample size and different species traits. Fish Res.

[47] Qi Y (2012). Random forest for bioinformatics.

[48] Alves-Bezerra M, Cohen DE (2017). Triglyceride metabolism in the liver. Compr Physiol.

[49] Wang C, Hu M, Yi Y, Wen X, Lv C, Shi M (2022). Multiomic analysis of dark tea extract on glycolipid metabolic disorders in db/db mice. Front Nutr.

[50] Marangoni F, Agostoni C, Borghi C, Catapano AL, Cena H, Ghiselli A (2020). Dietary linoleic acid and human health: focus on cardiovascular and cardiometabolic effects. Atherosclerosis.

[51] Mousavi SM, Jalilpiran Y, Karimi E, Aune D, Larijani B, Mozaffarian D (2021). Dietary intake of linoleic acid, its concentrations, and the risk of type 2 diabetes: a systematic review and dose-response meta-analysis of prospective cohort studies. Diabetes Care.

[52] Wang B, Wu L, Chen J, Dong L, Chen C, Wen Z (2021). Metabolism pathways of arachidonic acids: mechanisms and potential therapeutic targets. Signal Transduct Target Ther.

[53] Chawengsub Y, Gauthier KM, Campbell WB (2009). Role of arachidonic acid lipoxygenase metabolites in the regulation of vascular tone. American journal of physiology. Heart Circ Physiol.

[54] Imig JD (2012). Epoxides and soluble epoxide hydrolase in cardiovascular physiology. Physiol Rev.

[55] Deng Y, Theken KN, Lee CR (2010). Cytochrome p450 epoxygenases, soluble epoxide hydrolase, and the regulation of cardiovascular inflammation. J Mol Cell Cardiol.

[56] Zha W, Edin ML, Vendrov KC, Schuck RN, Lih FB, Jat JL (2014). Functional characterization of cytochrome p450-derived epoxyeicosatrienoic acids in adipogenesis and obesity. J Lipid Res.

[57] Luria A, Bettaieb A, Xi Y, Shieh G, Liu H, Inoue H (2011). Soluble epoxide hydrolase deficiency alters pancreatic islet size and improves glucose homeostasis in a model of insulin resistance. Proc Natl Acad Sci USA.

[58] Lettieri-Barbato D, Aquilano K (2020). Aging and immunometabolic adaptations to thermogenesis. Ageing Res Rev.

[59] Horvath S, Raj K (2018). DNA methylation-based biomarkers and the epigenetic clock theory of ageing. Nat Rev Genet.

[60] Sayed N, Huang Y, Nguyen K (2021). An inflammatory aging clock (iAge) based on deep learning tracks multimorbidity, immunosenescence, frailty and cardiovascular aging. Nat Aging.

[61] Rhoads TW, Anderson RM (2021). Taking the long view on metabolism. Science.

[62] Amorim JA, Coppotelli G, Rolo AP, Palmeira CM, Ross JM, Sinclair DA (2022). Mitochondrial and metabolic dysfunction in ageing and age-related diseases. Nat Rev Endocrinol.

[63] Kolb H, Kempf K, Martin S (2023). Insulin and aging—a disappointing relationship. Front Endocrinol.

[64] Sachiko Araki MOSG (2004). Impaired lipid metabolism in aged mice as revealed by fasting-induced expression of apolipoprotein mrnas in the liver and changes in serum lipids. Gerontology.

[65] Duan J, Liu J, Ruan B, Ding J, Fang Z, Xu H (2023). Age-related liver endothelial zonation triggers steatohepatitis by inactivating pericentral endothelium-derived c-kit. Nature aging.

[66] Bartelt A, Heeren J (2014). Adipose tissue browning and metabolic health. Nat Rev Endocrinol.

[67] Scheja L, Heeren J (2016). Metabolic interplay between white, beige, brown adipocytes and the liver. J Hepatol.

[68] Tchernof A, Després JP (2013). Pathophysiology of human visceral obesity: an update. Physiol Rev.

[69] Neeland IJ, Ross R, Després JP (2019). Visceral and ectopic fat, atherosclerosis, and cardiometabolic disease: a position statement. Lancet Diabetes Endocrinol.

[70] Johnson AA, Stolzing A (2019). The role of lipid metabolism in aging, lifespan regulation, and age-related disease. Aging Cell.

[71] Song R, Hu M, Qin X, Qiu L, Wang P, Zhang X (2023). The roles of lipid metabolism in the pathogenesis of chronic diseases in the elderly. Nutrients.

[72] Baker SA, Rutter J (2023). Metabolites as signalling molecules. Nat Rev Mol Cell Biol.

[73] Burns JL, Nakamura MT, Ma D (2018). Differentiating the biological effects of linoleic acid from arachidonic acid in health and disease. Prostaglandins Leukot Essent Fatty Acids.

[74] Mohallem R, Aryal UK (2020). Regulators of tnfα mediated insulin resistance elucidated by quantitative proteomics. Sci Rep.

[75] Huangfu L, Li R, Huang Y, Wang S (2023). The il-17 family in diseases: from bench to bedside. Signal Transduct Target Ther.

[76] Girbl T, Lenn T, Perez L, Rolas L, Barkaway A, Thiriot A (2018). Distinct compartmentalization of the chemokines cxcl1 and cxcl2 and the atypical receptor ackr1 determine discrete stages of neutrophil diapedesis. Immunity.

[77] De Filippo K, Dudeck A, Hasenberg M, Nye E, van Rooijen N, Hartmann K (2013). Mast cell and macrophage chemokines cxcl1/cxcl2 control the early stage of neutrophil recruitment during tissue inflammation. Blood.

[78] Sullivan DE, Ferris M, Nguyen H, Abboud E, Brody AR (2009). Tnf-alpha induces tgf-beta1 expression in lung fibroblasts at the transcriptional level via ap-1 activation. J Cell Mol Med.

[79] Thiel G, Rössler OG (2014). Resveratrol stimulates ap-1-regulated gene transcription. Mol Nutr Food Res.

[80] Zhu X, Yao Q, Yang P, Zhao D, Yang R, Bai H (2022). Multi-omics approaches for in-depth understanding of therapeutic mechanism for traditional chinese medicine. Front Pharmacol.

