# Supplementary figures and images for: Bazi Bushen ameliorates age-related energy metabolism dysregulation by targeting the IL-17/TNF inflammatory pathway associated with SASP
Source: Chin Med. 2024 Apr 9;19:61. doi: 10.1186/s13020-024-00927-9 (PMC11005220; doi:10.1186/s13020-024-00927-9)

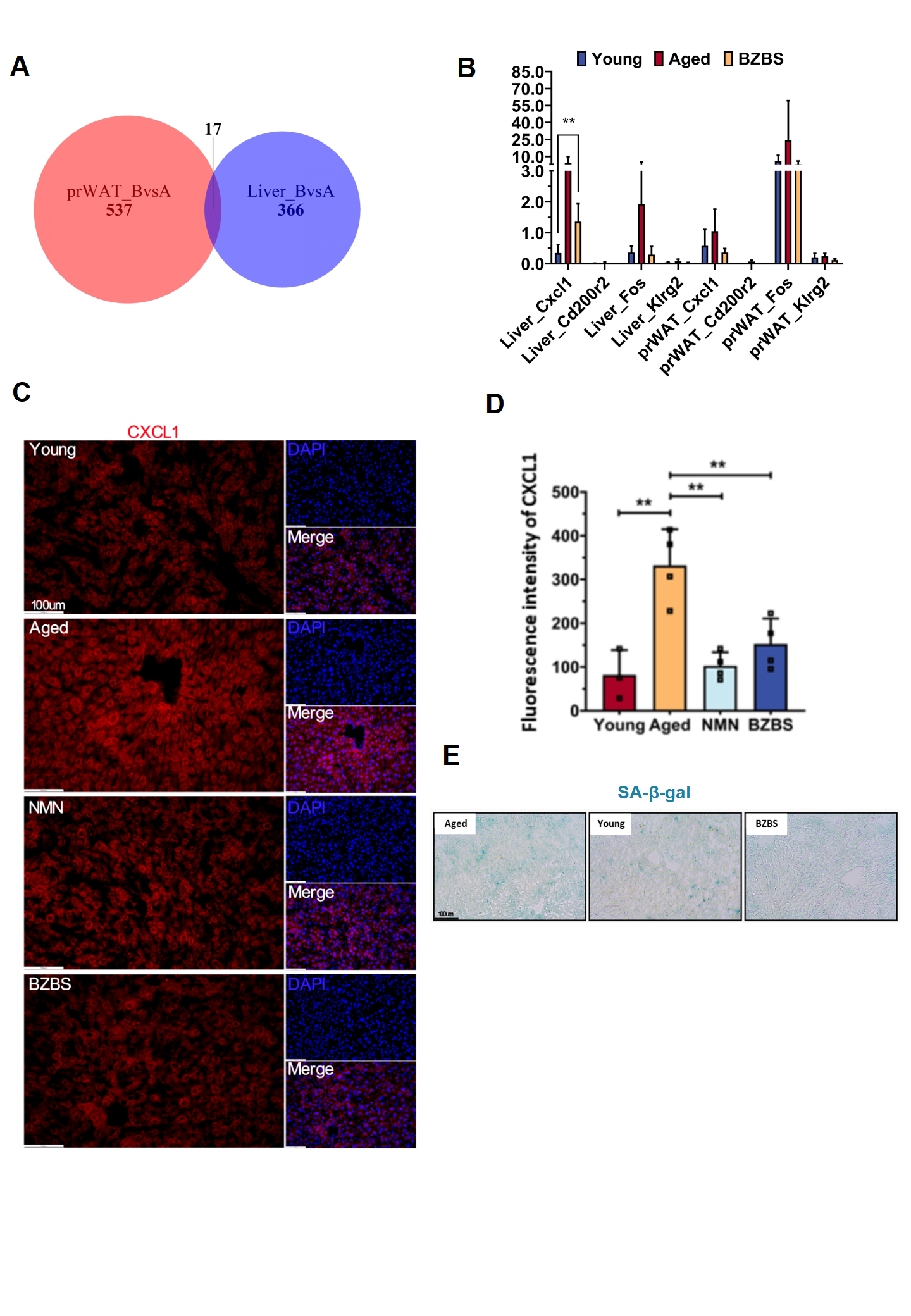

Supplement: Supplementary file 1 — Additional file 1: Figure S1. BZBS reduces liver senescence cells accumulation in mice. (A) The Venn diagram displays the intersection quantity of DEGs (Differentially Expressed Genes) between perirenal fat and liver BZBS vs. Aged (B vs A). (B) The bar chart shows the expression of intersection inflammation-related genes. (C) The Immunofluorescence of Cxcl1 in the mouse liver. (D) The aging-associated β-galactosidase staining of mouse liver. Data is presented as mean ± standard deviation. *p < 0.05, **p < 0.01. [file 13020_2024_927_MOESM1_ESM.tif]

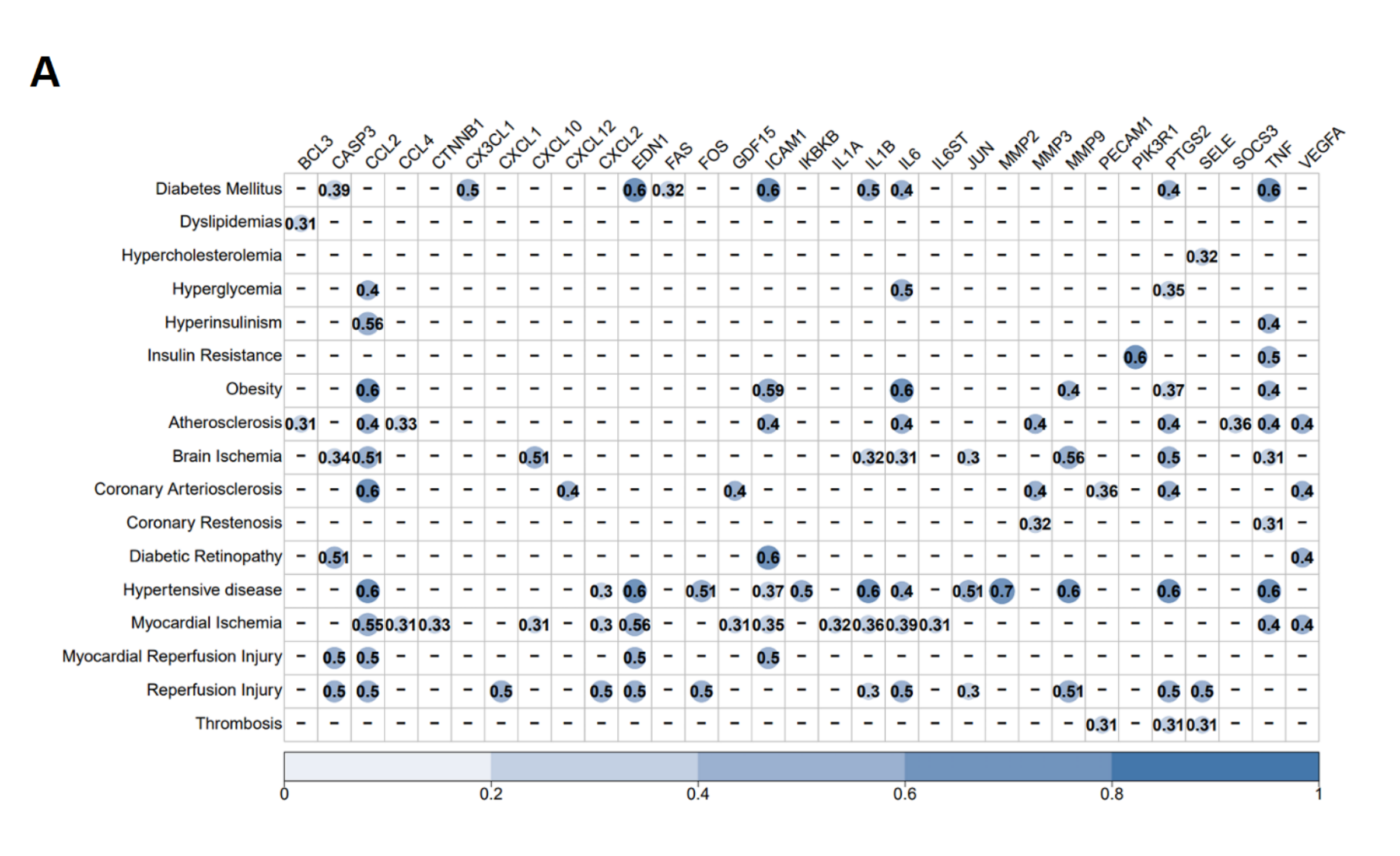

Supplement: Supplementary file 2 — Additional file 2: Figure S2. BZBS reduces susceptibility to age-related metabolic diseases. (A) Correlation analysis of inflammation-related genes in the TNF signaling pathway, IL-17 signaling pathway, and SASP pathway with age-related metabolic diseases in the BZBS and Aged comparison groups. [file 13020_2024_927_MOESM2_ESM.tif]

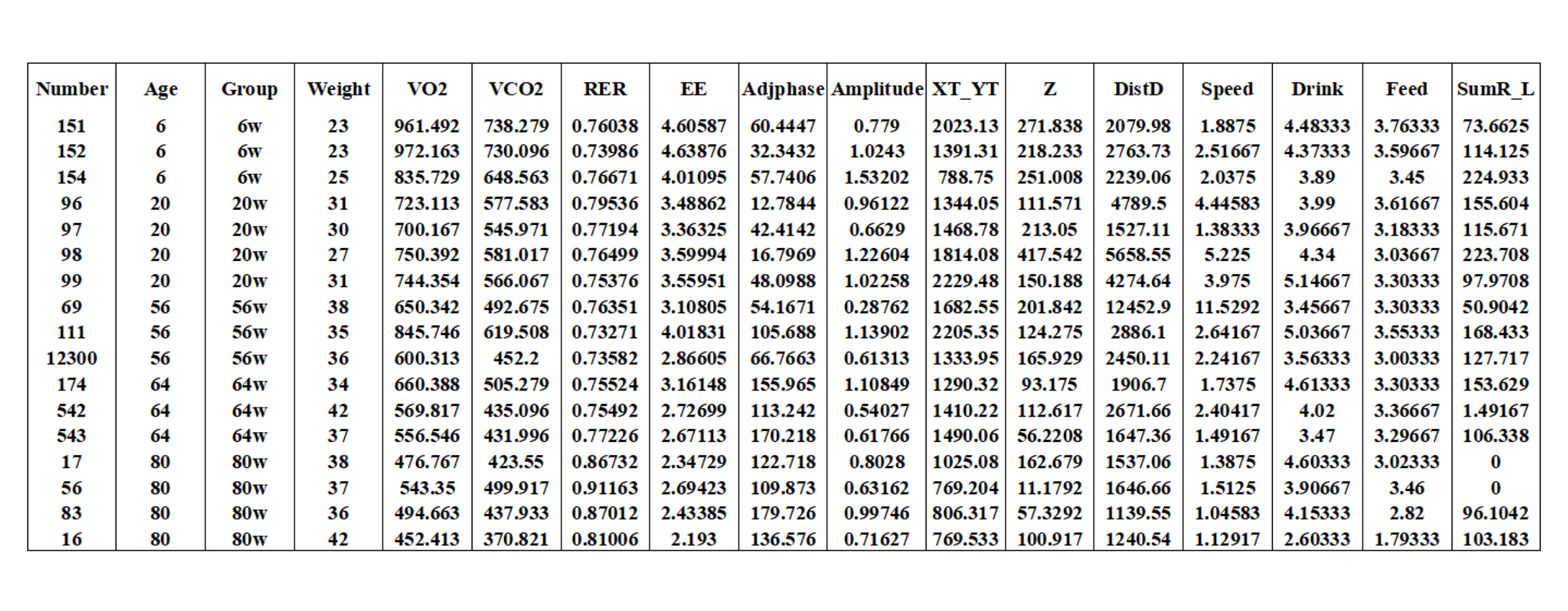

Supplement: Supplementary file 3 — Additional file 3: Table S1-S16. Raw data refer to table. [file 13020_2024_927_MOESM3_ESM.tif]
